# The role of visuomotor synchrony on virtual full‐body illusions in children and adults

**DOI:** 10.1111/jnp.12372

**Published:** 2024-05-09

**Authors:** Hayley Dewe, Oscar Sill, Simon Thurlbeck, Robert W. Kentridge, Dorothy Cowie

**Affiliations:** ^1^ Department of Psychology Durham University Durham UK; ^2^ Brain, Mind and Consciousness program Canadian Institute for Advanced Research (CIFAR) Toronto Ontario Canada

**Keywords:** body movement, body representation, children's development, embodiment, multisensory integration, skin conductance, virtual reality

## Abstract

The present study explored the effects of visuomotor synchrony in virtual reality during the embodiment of a full human avatar in children (aged 5–6 years) and adults. Participants viewed their virtual bodies from a first‐person perspective while they moved the body during *self‐generated* and *structured* movement. Embodiment was measured via questions and psychophysiological responses (skin conductance) to a virtual *body‐threat* and during both movement conditions. Both children and adults had increased feelings of ownership and agency over a virtual body during synchronous visuomotor feedback (compared to asynchronous visuomotor feedback). Children had greater ownership compared to adults during synchronous movement but did not differ from adults on agency. There were no differences in SCRs (frequency or magnitude) between children and adults, between conditions (i.e., baseline or movement conditions) or visuomotor feedback. Collectively, the study highlights the importance of visuomotor synchrony for children's ratings of embodiment for a virtual avatar from at least 5 years old, and suggests adults and children are comparable in terms of psychophysiological arousal when moving (or receiving a threat to) a virtual body. This has important implications for our understanding of the development of embodied cognition and highlights the considerable promise of exploring visuomotor VR experiences in children.

## INTRODUCTION

To navigate within our environment and to interact with others or objects around us is a fundamental part of human existence and requires a coherent sense of *embodiment*. Embodiment refers to feeling ‘present’ and located within the physical body (Gallagher, 2000). It is characterised by sensations of ownership (i.e., feeling that a body belongs to you) and agency (i.e., feeling that you can control its movement). Stable embodiment is reliant on the binding of multisensory information (e.g., visual, tactile, proprioceptive) to body parts or – in bodily illusions – to an external object (Ehrsson, 2012). Embodiment typically arises when incoming multisensory signals are congruent with internal prior knowledge or expectation about the body or its action (Ehrsson, 2012; Seth et al., 2012).

One case of this is in the Rubber Hand Illusion (RHI: Botvinick & Cohen, 1998), where synchronous visuotactile stimulation of the participant's own (hidden) hand and a rubber hand elicits feelings of ownership for the rubber hand. Much of this research has been conducted with adult samples; however, some research suggests children aged 4–9 years show adult‐like effects of synchronous visuotactile stimulation on their feelings of ownership for the rubber hand (Cowie et al., 2013; Filippetti & Crucianelli, 2019). However, it is accompanied by a heavy reliance on visual signals compared to adults, regardless of visuotactile synchrony (Cowie et al., 2013, 2016; Filippetti & Crucianelli, 2019).

This visual reliance is broadly in line with what we know of children's broader developing body representations. These are central to both their interactions with their environment and their emerging sense of self. In terms of interacting with the environment and controlling the actions within it, for children visual information dominates the other senses. By 4–5 years of age, children have learned through copious visuomotor experience to correctly judge the fit between their felt body and visually specified obstacles in the external world (Adolph et al., 2018); to weigh visual cues more highly than adults when estimating hand location (Cowie et al., 2013; von Hofsten & Rösblad, 1988); and to adopt a visual reference frame for processing touches to the body (Begum Ali et al., 2014). In terms of the emerging sense of self, classic mirror self‐recognition tasks show that by around 2 years, children possess an explicit sense of bodily awareness (Lewis, 2011). During mid‐childhood, some refinement takes place in how visuotactile cues are used to establish an explicit awareness of body ownership (Cowie et al., 2018, 2022). However, rather little is known about how dynamic visuomotor signals underpin this explicit understanding of self; or how they might scaffold the affective, defensive reactions to bodily threats, which are a hallmark of bodily self‐awareness (de Vignemont, 2018).

In bodily illusions, embodiment is typically quantified via questionnaire ratings (e.g., agreement on how much the virtual body feels like one's own) or more objective measures, such as skin conductance responses (SCRs) of the electrodermal activity complex (EDA) which measure autonomic (emotional) responding to stressful or fearful situations when the body is perceived to be threatened. A wealth of studies (notably with adults) has found increased autonomic responding (SCRs) to perceived body‐threats to the rubber hand (e.g., Armel & Ramachandran, 2003) or a virtual hand/body (Ehrsson, 2007; Petkova & Ehrsson, 2008) after synchronous visuotactile conditions only, as opposed to asynchronous (incongruent) conditions. Collectively, it suggests synchronous multisensory integration (e.g., visuotactile signals) is crucial for embodiment for an external limb/body and reflected by discrete changes in autonomic psychophysiological arousal. While informative, much of this research is based on static paradigms that do not include a moving body/limb.

### The importance of movement

Movement provides us with rich multisensory cues about the body and is fundamental in driving embodiment at all ages. Already by 3 months, infants explore their surroundings through congruent movement, e.g., kicking a mobile (Rovee‐Collier, 1999), and the cause‐effect relation between felt body movement and viewed kicks may generate agency and inform them about their own bodies (Zaadnoordijk et al., 2018). Likewise, from research on preferential‐looking paradigms, we know infants can detect visuomotor correlations between their own movement and the synchronous movement of legs viewed on a screen (Bahrick & Watson, 1985; Morgan & Rochat, 1997; Rochat, 1998). However, further development in the use of these visuomotor signals is likely. The intersensory coordination of movement (e.g., reaching) increases during infancy, with vision becoming more integrated into tactile and proprioceptive signals (Chinn et al., 2019). Children have less experience interacting with their bodies, which are also constantly changing and developing (de Klerk et al., 2021). Therefore, while fundamental sensory building blocks are present in the first few years of life, it is not yet clear how they contribute to explicit representations of the body during childhood.

To explore the role of synchronous movement on embodiment, researchers have used virtual versions of the RHI and a full‐body illusion (FBI). In the FBI, individuals can embody a whole virtual body (Slater et al., 2010). Typically, the illusion occurs from synchronous visuomotor or visuotactile feedback, and a first‐person observer perspective (Ehrsson, 2007; Kokkinara et al., 2016; Kondo et al., 2020; Lenggenhager et al., 2007). The principal findings from this research with adult samples are significant increases in embodiment (e.g., ownership) for a virtual avatar when an avatar moves synchronously with the participant's own movements (Maselli & Slater, 2013; Peck et al., 2013; Sanchez‐Vives et al., 2010; Sanchez‐Vives & Slater, 2005; Slater et al., 2010). However, many of these studies have focused on adult samples. There is still little understanding of how these visuomotor processes can drive bodily illusions across childhood. Cowie et al. (2018) found children aged 6–9 years reported an adult‐like increased ownership of the virtual body when visuotactile stimulation was synchronous rather than asynchronous. However, the difference between synchronous and asynchronous stroking conditions increased significantly with age, suggesting children have a relatively long developmental trajectory of refining how they use multisensory information for embodiment. Using a visuomotor version of the RHI, Dewe et al. (2021) found children aged 4–14 years felt increased feelings of embodiment (ownership and agency) during visuomotor synchrony (when the virtual hand matched the participant's own movements) compared to asynchronous movement.

In addition, two studies looked directly at FBI in children and adults. Keenaghan et al. (2022) used the FBI where 5‐year‐olds and adults embodied different‐sized bodies during visuomotor synchrony and asynchrony. In contrast to adults, children reported sensations of ownership and agency for the virtual body regardless of the visuomotor feedback condition. Similarly, Weijs et al. (2021) found visuomotor synchrony modulated feelings of ownership, but not agency during a FBI in 8–12‐year‐olds. Together, with the findings from visuotactile research (that the effect of synchrony increases with age: Cowie et al., 2018), this could imply the effects of visuomotor synchrony are only prevalent in embodiment from at least 8 years old, while 5‐year‐olds still rely on visual cues for embodiment. The existing body of literature has yet to provide a cohesive explanation of the development, and constraints of these processes in children – particularly in relation to virtually moving bodies.

### The present study

The present study sought to address the inconsistencies and augment the limited body of literature by exploring children's visuomotor cues on embodiment using the FBI. We compared adults’ and children's (aged 5–6 years) levels of embodiment under conditions of synchronous and asynchronous visuomotor feedback. Children of 5–6 years were studied to provide a direct comparison with the previous developmental work on visuomotor full‐body illusions conducted by Keenaghan et al. (2022) and Weijs et al. (2021), enabling us to establish how visuomotor cues drive embodiment at this age. In line with previous research, we created an enticing experiment for young children by using a version of the ‘Alice in Wonderland garden tea party’ (Keenaghan et al., 2022), and selected parameters known to generate high sensations of embodiment, i.e., participants viewed the body from a first‐person perspective (Kokkinara et al., 2016) and could see themselves in a virtual mirror (Preston et al., 2015; Slater et al., 2010). Measures of embodiment were quantified using established questions suitable for children (Dewe et al., 2021; Keenaghan et al., 2022) and objective psychophysiological responses for a virtual body‐threat (Weijs et al., 2021). In contrast to previous research with adults (e.g., knife threats; Petkova & Ehrsson, 2008), we designed a more child‐friendly body‐threat of snowballs directed towards the virtual body to suit young children.

We implemented a structured movement period for more control and consistent limb movements to be studied across age groups and conditions where simple tasks such as reaching in VR have been reliably used in VR embodiment studies with children and adults (Dewe et al., 2021; Keenaghan et al., 2022; Weijs et al., 2021). Notably, we also included a self‐generated, undirected free movement period, in which participants could move the body independently. Crucially, this allowed us to explore embodiment after self‐generated and structured (task‐directed) motor experience. Research suggests that free active movement can improve perceived ownership and ‘presence’ in virtual reality compared to instructed movement (Choi et al., 2016), and self‐generated action plays an important role in sensations of agency and the perception of one's body (Tsakiris & Haggard, 2005). We hypothesised that children would show adult‐like preferences of visuomotor synchrony driving embodiment for a virtual avatar (Dewe et al., 2021; Weijs et al., 2021), and threat‐SCRs would be greater during visuomotor conditions. Notably, Weijs et al. (2021) did not find threat responses to be modulated by experimental conditions. Whether differences in SCRs will be observed for a younger sample of children, or under specific visuomotor conditions is yet to be considered.

## MATERIALS AND METHODS

### Participants

For this study, we recruited 17 children (5 females) aged 5–6 years (*M* = 6 years, SD = .64) and 20 adults (15 females) aged 18–26 years (*M* = 20 years, SD = 1.71) from the North East of England, UK. Children were recruited through a volunteer participant pool, and adults through the Department of Psychology at Durham University. Exclusion criteria and pre‐screening ensured all participants had normal or corrected‐to‐normal vision, no known neurodevelopmental differences, and no motor impairments that would affect their ability to take part in the virtual reality experiment.

### Design

We used a mixed design to compare the responses of children and adults (between‐subjects) and each participant during both synchronous and asynchronous movement conditions (within‐subjects). The order of presentation of synchronous and asynchronous conditions was counterbalanced across participants by alternating the starting condition on each occasion. In the synchronous condition, the virtual body moved synchronously with the participant's own body. In asynchronous movement conditions, each participant viewed pre‐recorded videos of themselves moving around in an initial ‘practice run’ recorded during the experimental setup.

### Materials

**FIGURE 1:**
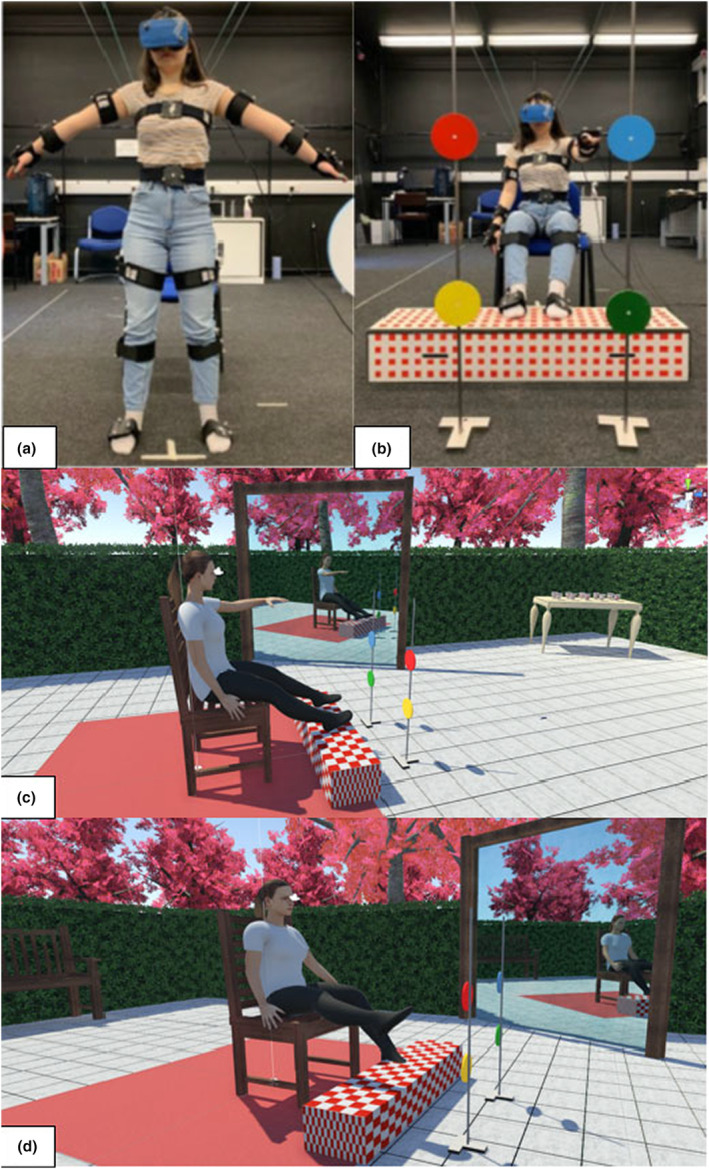
Set up images of the virtual reality experiment. (a) an adult participant wearing the motion capture clusters on the arms torso, legs, feet, and the Oculus Rift headset; (b) real‐world environment of the footrest and coloured targets, (c) the virtual avatar in the ‘Alice in Wonderland garden tea party’ during the free movement task (looking in the mirror) and (d) the virtual avatar moving during a structured movement (reaching) task, where the participant is reaching their leg towards the red target.

All participants were placed into a virtual environment where they viewed a full virtual body (avatar) from a first‐person perspective (Figure 1 and ‘Procedure Video’ in Supplementary material). The virtual reality laboratory (Department of Psychology, Durham University) has a fully integrated system fitted with 16 x Vicon Bonita infrared cameras (Vicon, Oxford UK) connected to *Vicon Tracker* software (3.6.1) and *Vicon Pegasus* software (Pegasus 1.2.1) to track and map the participant's movement onto a corresponding realistic virtual avatar. The infrared cameras were calibrated to 14 reflective clusters worn by the participants (via straps, Figure 1a), and five markers were fitted to an Oculus Rift headset (Oculus Consumer Version; Menlo Park, CA, USA).

In the virtual environment, participants viewed a custom‐built ‘Alice in Wonderland’ themed garden tea party (similar to Keenaghan et al., 2022) developed in Unity (Unity Technologies, San Francisco, CA, USA), which featured replicas of objects such as the chair and coloured targets for reaching (Figure 1b,c). We measured the participant's height, and distance from the chair to the colour targets and footrest to match their body location and position in the virtual world. Virtual avatars were pre‐set designs (male/female, adult/child) developed in MakeHuman software (https://www.makehumancommunity.org) that matched participants in sex and age, but no other characteristics (avatars had fair skin and wore simple clothing). Participants remained seated for the entire experiment for safety and greater consistency across tasks.

Participants rated their level of embodiment for the virtual body with a question on ownership and agency; they also rated a control statement (Table 1). These were based on previously established measures (Gonzalez‐Franco & Peck, 2018), which have similarly been used with young children (Cowie et al., 2013, 2016; Dewe et al., 2021). Each item was rated on agreement using a 7‐point Likert scale ranging from 0 (“No, definitely not”) to 7 (“Yes lots and lots”). Questions were answered twice, after each visuomotor feedback condition and in a randomised order across participants.

**TABLE 1:** Embodiment questionnaire items.

	Question	Component
Q1	When I was in the virtual scene, sometimes it felt like I was controlling the body that I saw	Agency
Q2	When I was in the virtual scene, sometimes it felt like the body I saw was my own body	Ownership
Q3	When I was in the virtual scene, sometimes it felt like I was growing a tail	Control

### Procedure

Participants were told they would visit a virtual ‘Alice in Wonderland garden tea party’. The expectation of a body‐threat was introduced at the start, with the experimenter stating the “*Mad Hatter might be hiding to throw some snowballs at you*”. After obtaining verbal assent from the children, and written consent from the children's guardian/parent and the adult participants, participants were randomly assigned to an order of movement conditions and had their height measured as detailed earlier. They were fitted with SCR electrodes to their left hand, motion capture clusters to the body, and VR headset. The experimenter calibrated the clusters in *Vicon Pegasus* software to map the participant's body onto movements of the virtual avatar. All participants practised listening to movement instructions, moved the virtual body to practice reaching for the targets, and told how to use the Likert scales for the questions. This training ‘practice run’ was later played back during asynchronous movement trials. The experiment then began, consisting of four tasks: (i) baseline period (ii) free movement period, (iii) structured movement task, and (iv) body‐threat task (see “Procedure Video” in Supplementary materials).

In the *baseline period*, participants observed a plain cross in the virtual environment for 90 s. This allowed SCR measures to stabilise, and generated a baseline level of SCR responding which was used to standardise the later event‐related SCRs. Participants then entered the virtual tea party, where they completed the *free movement period* for 60 s during which they were instructed to “move around freely and explore the body however you like but remain sitting down”. This encouraged participants to move freely, observing it from a first‐person perspective or using the virtual mirror.

Following this, participants completed the *structured movement task* for 60 s. This was a controlled, consistent task where they followed instructions to reach for the coloured targets with their hands and feet. The instructions, given every 6 s via external laboratory speakers and computerised speech software, were a simple list of target instructions (e.g., “hand‐red”). In the asynchronous condition, participants followed these instructions while watching their own pre‐recorded movements, where they followed instructions in a different order.

Participants were instructed to remain still and look ahead, while the *body‐threat* appeared and moved towards the body. This was a cluster of three snowballs travelling towards and through the torso of the virtual body. They each appeared in the bush in front of the participant and travelled at medium speed (*y* = *x* ^3^ where *y* is displacement in metres and *x* is time in seconds) for 2.80 s. They had a 1‐s visible trail feature showing the snowball's previous trajectory, to help indicate speed. During this period autonomic arousal (SCR) was recorded for 8 s^1^ from threat onset to catch all potential responses tied to the threat stimulus.

After this, participants exited virtual reality and answered the embodiment questions on the experience they had just received. Next, participants re‐entered virtual reality to complete the free movement, structured movement, and body‐threat tasks again in the remaining visuomotor condition (synchronous or asynchronous), and again answer the embodiment questions for this second experience. At the end of the session, participants had markers and electrodes removed and were debriefed and thanked. The entire session including setup lasted approximately 1 h.

### SCR analysis

Autonomic arousal was quantified via objective psychophysiological measures of electrodermal activity: skin conductance responses (SCRs) in all experimental tasks including stimulus‐specific SCRs to the presentation of a body‐threat (i.e., SCRs induced by the presentation of the snowball threat). We also investigated SCRs (frequency and magnitude) during the baseline, free movement, and structured movement periods as measures of spontaneous autonomic arousal unrelated to a threat, which could provide information on an individual's background autonomic activity levels and SCR profile while embodying a full body avatar.

All SCR data were collected using an MP160 Biopac unit (Biopac Systems Inc., Goleta, CA), with SS57L sensor leads and pre‐gelled Ag‐AgCl electrodes (EL507) attached to the index and middle distal phalanges of the participant's left hand. The data acquisition rate was 2000 Hz and SCRs were defined as a delta (difference) function between the onset of a signal deflection from the background (tonic) to the maximum amplitude (peak) of the deflection reached (Braithwaite et al., 2013). The threshold criteria for SCR onset was set to the signal crossing a threshold of .01 μS (microsiemens) based on established guidelines (Boucsein, 2012; Braithwaite et al., 2013). Data signals were first visually analysed for artefacts or noise and artefacts, before being analysed using *Biopac AcqKnowledge* (v4.2).

For body‐threat SCRs, the average magnitude values were calculated to consider the frequency/rate of responding (i.e., to include zero responses). Threat‐related SCRs were defined as the largest SCR occurring during an 8‐s window after the cluster snowballs were fired (Braithwaite et al., 2013). Based on established recommendations all SCRs magnitudes were normalised via Log (SCR + 1) transformations and standardised via *z*‐score transformations to facilitate individual difference analysis (Braithwaite et al., 2013). To do this, SCRs from all experimental conditions (i.e., the baseline, free movement, structured movement, and body‐threat) were pooled for each participant to generate a representative and average sample of SCRs for that individual. This ensured SCR magnitudes for a specific task were an accurate representation of their capacity and parameters of general autonomic responsivity.

## RESULTS

We first present the questionnaire data followed by the SCR data, on both the body‐threat and movement periods in a virtual body. The questionnaire analysis is based on the whole sample of 17 children (Mean age = 6, SD = .66) and 20 adults (Mean age = 20, SD = 1.71). For the SCR analysis, four participants (two children and two adults) were removed from the whole sample who were classified as hypo‐responders (Boucsein, 2012; Braithwaite et al., 2013; Dawson et al., 2007). This was based on a set criterion of a minimum of 6 SCRs (at least 1 SCR per minute) across the entire experimental procedure (total time 5.5 min). This resulted in a sample of 15 children (Mean age = 6, SD = .64) and 18 adults (Mean age = 20, SD = 1.80) for all SCR analyses.

Where applicable, non‐parametric tests were applied for non‐normally distributed data (e.g., questionnaires) and adjusted values were taken for violations of homogeneity. Post‐hoc comparisons were analysed via independent *t*‐tests for parametric analysis, and Mann–Whitney *U* tests for non‐parametric analysis. Adjusted Bonferroni (*p*_*bonf*_) values were used to correct for multiple Post hoc comparisons. Effect sizes are provided as partial eta squared ($\eta_{p}^{2}$) and the rank‐biserial correlation (*r*_B_). We also conducted Bayesian analyses (Bayes Factor BF_10_) in JASP v0.18.1 (JASP Team, 2023) to indicate the probability of the data being in favour of the alternative or null hypothesis (Quintana & Williams, 2018).

### Questionnaires

Before analysing the embodiment questions, we checked responses to the control question for synchronous and asynchronous movement conditions to determine if there were any differences in questionnaire understanding or bias in answers between the children and adults. Generally, responses to this question were low (0 = indicating no tail) in both movement conditions for children (both synchronous and asynchronous: Mdn = 0, IQR = 0) and adults (both synchronous and asynchronous: Mdn = 0, IQR = 0). Mann–Whitney *U* tests revealed no significant difference between groups on the Control question for either the synchronous (*U* = 195, *p* = .244, *r*_B_ = .15, BF_10_ = .48) or asynchronous (*U* = 186, *p* = .442, *r*_B_ = .09, BF_10_ = .43) conditions. Wilcoxon signed‐rank *t*‐tests revealed no effect of movement synchrony on the Control question for adults (*Z* = 1.342, *p* = .346, *r*_B_ = 1.00, BF_10_ = .31) or children (*Z* = .447, *p* = 1.00, *r*_B_ = .33, BF_10_ = .37). The BF indicates anecdotal (slight) evidence that the groups are comparable. However, given that most children answered correctly (‘no’) to this question^2^ we are confident that the young children answered appropriately and reliably compared to adults and any observable differences between age or condition are unlikely due to differences in understanding or response bias.

#### Agency

**FIGURE 2:**
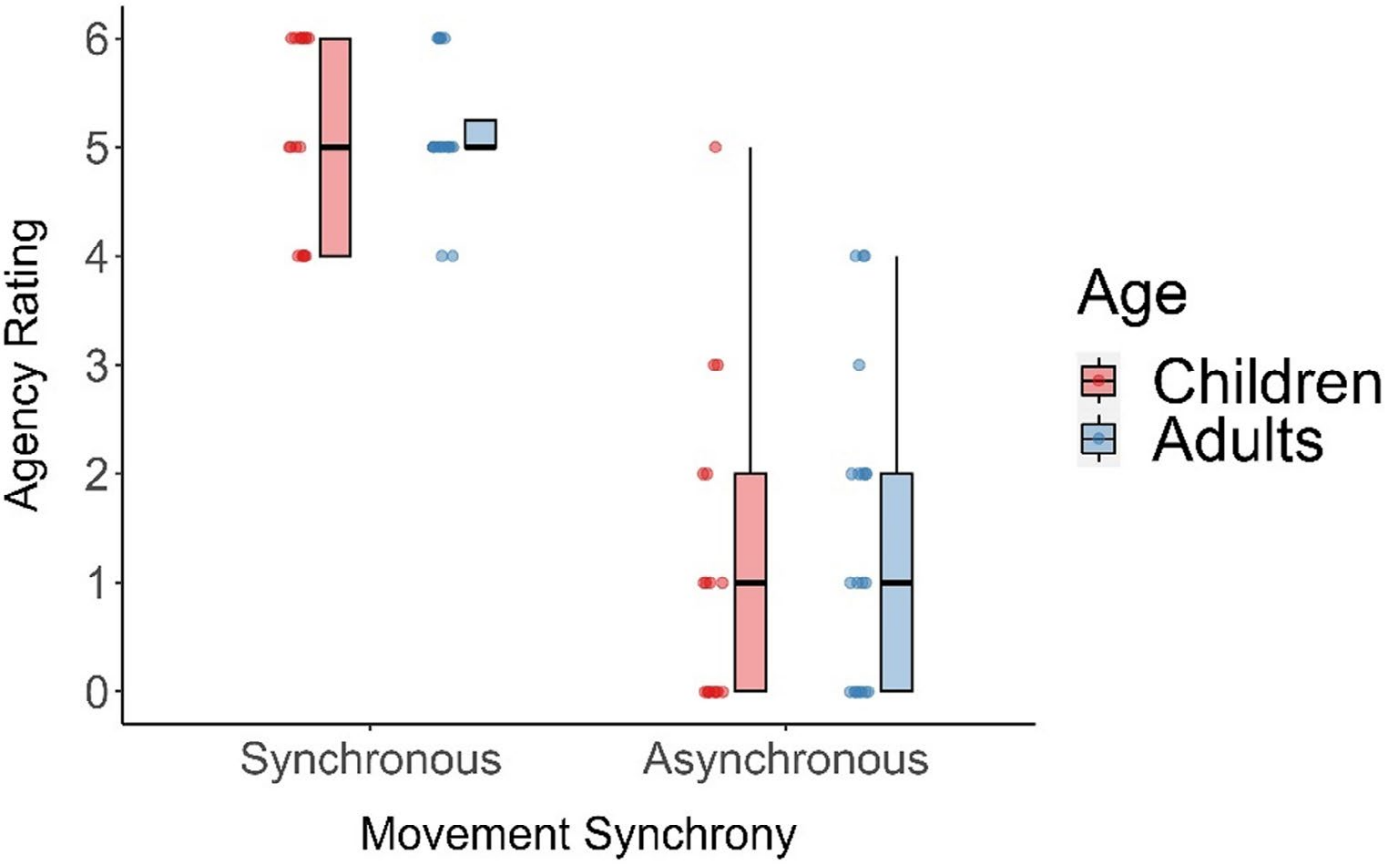
Average agency ratings for the synchronous and asynchronous visuomotor feedback conditions for adults and children.

Average agency ratings for synchronous and asynchronous visuomotor feedback were high/above the median for both groups (Figure 2). Mann–Whitney *U* tests revealed no significant difference between groups on agency ratings for the synchronous (*U* = 179, *p* = .779, *r*_B_ = .05, BF_10_ = .35) or asynchronous (*U* = 144, *p* = .417, *r*_B_ = .15, BF_10_ = .39) conditions. The BF values suggest anecdotal (slight) evidence that the groups are similar. Within groups, Wilcoxon signed‐rank *t*‐tests revealed increased agency ratings for synchronous compared to asynchronous conditions for adults (*Z* = 3.920, *p* < .001, *r*_B_ = 1.00, BF_10_ = 538.79) and children (*Z* = 3.621, *p* < .001, *r*_B_ = 1.00, BF_10_ = 670.40). The BF values indicate decisive evidence, which suggests increased feelings of agency during synchronous visuomotor feedback compared to asynchronous visuomotor feedback in both groups.

#### Ownership

**FIGURE 3:**
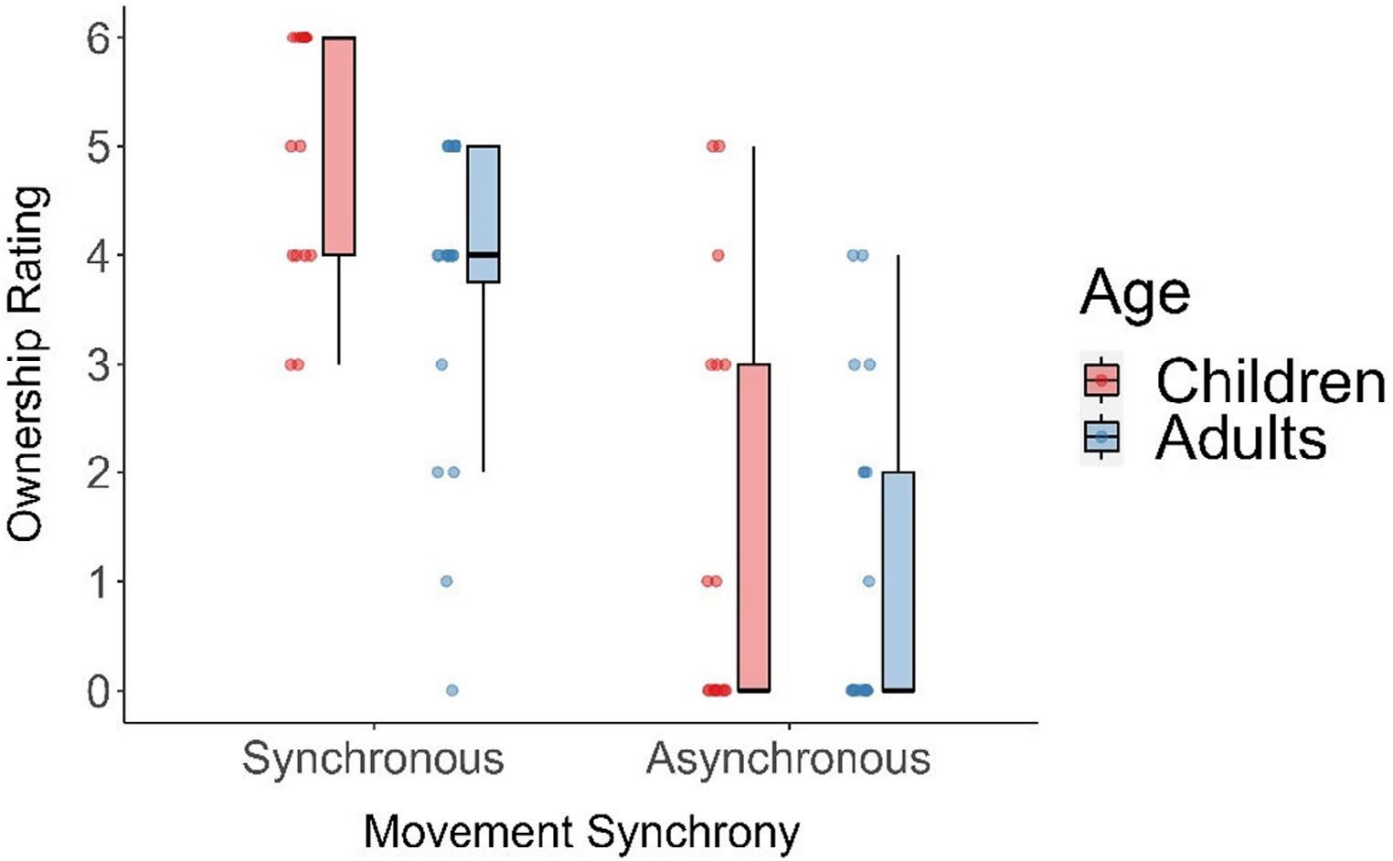
Average ownership ratings for the synchronous and asynchronous visuomotor feedback conditions for adults and children.

Average ownership ratings for synchronous and asynchronous visuomotor feedback were also high/ above the median (Figure 3). Mann–Whitney *U* tests revealed that children had significantly higher Ownership ratings compared to adults during the synchronous condition (*U* = 258, *p* = .006, *r*_B_ = .52, BF_10_ = 6.66), but not for the asynchronous (*U* = 189, *p* = .532, *r*_B_ = .11, BF_10_ = .42) condition. Here, BF values indicate substantial evidence that children had increased ownership compared to adults during synchronous movement (and anecdotal evidence the groups were similar in asynchronous movement). Wilcoxon signed‐rank t‐tests also revealed that ownership ratings were significantly higher during synchronous movement compared to asynchronous movement for both adults (*Z* = 3.823, *p* < .001, *r*_B_ = 1.00, BF_10_ = 958.44) and children (*Z* = 3.621, *p* < .001, *r*_B_ = 1.00, BF_10_ = 984.28). The BF values indicate decisive evidence that both adults and children had increased feelings of ownership during synchronous visuomotor feedback compared to asynchronous visuomotor feedback.

### Autonomic (emotional) arousal (SCRs) to a virtual body threat

#### Task efficacy: Threat SCR frequency

First, we analysed the efficacy (frequency) of the body‐threat (snowballs) in eliciting a fear response. The fear response was defined as the largest threat‐SCR occurring within an 8‐s window after the threat presentation. The body‐threats generated a threat‐response in 60% of children in synchronous movement and 67% of children during asynchronous movement. Whereas the body‐threat elicited a threat‐response in 56% of adults in the synchronous movement condition, and 39% of adults in the asynchronous movement condition. Mann–Whitney *U* tests revealed no significant difference between groups on threat‐SCR frequency during synchronous (*U* = 141, *p* = .816, *r*_B_ = .04, BF_10_ = .38) or asynchronous (*U* = 173, *p* = .122, *r*_B_ = .28, BF_10_ = .60) movement. Wilcoxon signed‐rank tests revealed no difference between the movement conditions in eliciting a threat‐SCR for either group (all *p* > .299, *r*_B_ < .43, BF_10_ = .33–.41). The BF values suggest anecdotal evidence that SCR frequency was comparable between groups and within movement conditions.

#### Autonomic responding: Threat SCR magnitudes

**FIGURE 4:**
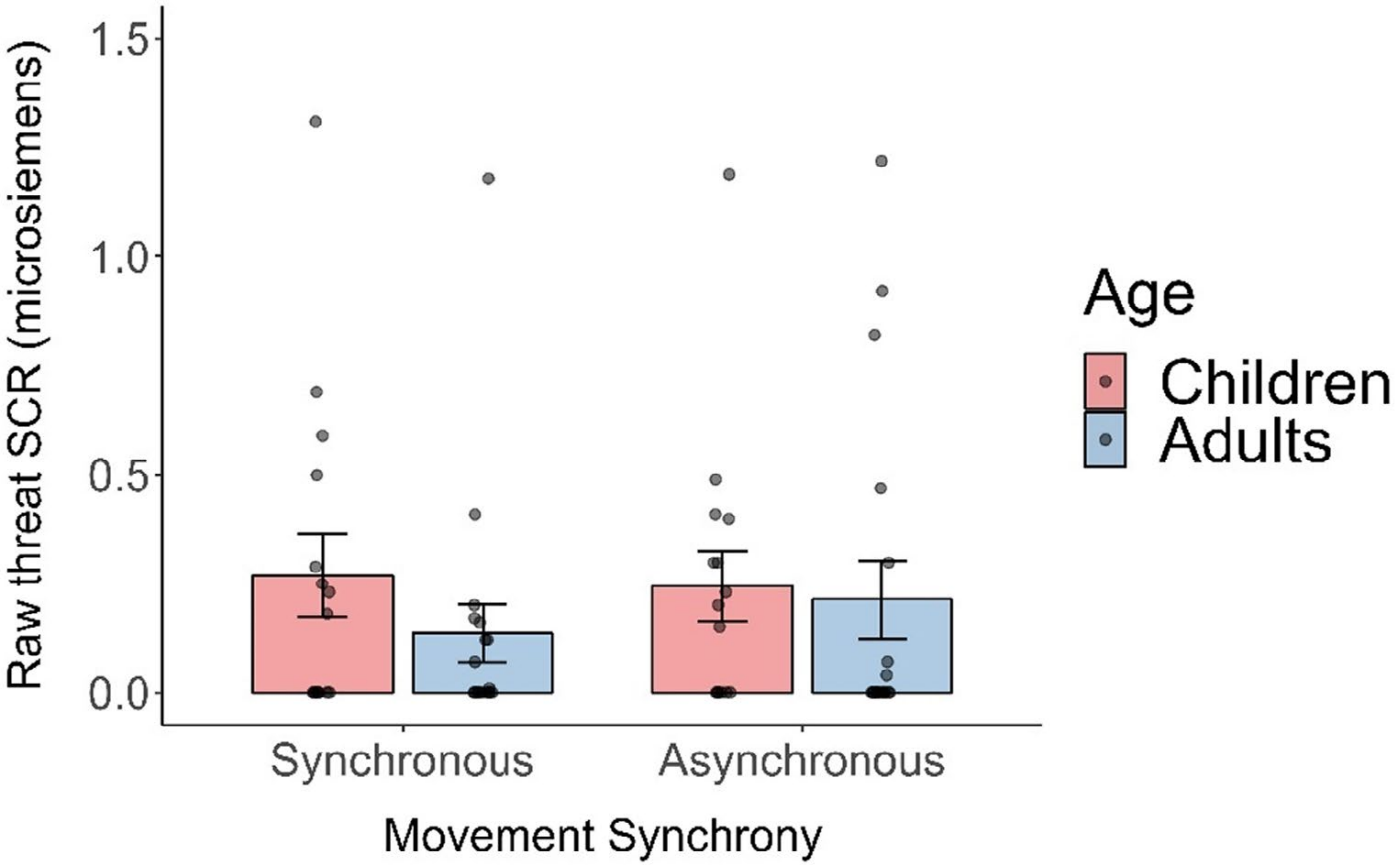
Average threat‐SCRs (raw microsiemens) for the synchronous and asynchronous visuomotor feedback conditions for adults and children.

We measured the average magnitude of standardised (*z*‐scored) threat‐SCRs for each movement condition. The raw (non‐standardised) threat‐SCR magnitudes are presented in Figure 4 (see Table 3 for standardised responses). A 2 (movement: synchronous, asynchronous) × 2 (group: children, adults) mixed ANOVA revealed no main effect of synchrony, *F*(1, 31) = .263, *p* = .612, $\eta_{p}^{2}$ = .008, BF_10_ = .29, no main effect of group, *F*(1, 31) = 1.285, *p* = .266, $\eta_{p}^{2}$ = .040, BF_10_ = .64 and no significant interaction *F*(1, 31) = .007, *p* = .932, $\eta_{p}^{2}$ = 2.382 × 10^−4^, BF_10_ = .32. The BF values mainly suggest substantial evidence that there was no difference between children and adults in terms of threat‐SCR magnitudes to a perceived body‐threat, and it was not affected by visuomotor feedback for either group.

### Autonomic arousal (SCRs) when moving a virtual body

In addition to the perception of a body‐threat directed at the body, participants experienced the virtual body during a free movement task and a structured (targeted) reaching task under conditions of both synchronous and asynchronous visuomotor feedback. We analysed the frequency and average magnitude (strength) of SCRs to indicate general levels of autonomic arousal during these movement tasks compared to the baseline period, where participants remained still and had no VR experience.

#### Frequency of elicited SCRs

The frequency of elicited SCRs during the five condition phrases: baseline, free movement (synchronous and asynchronous), and structured movement (synchronous and asynchronous) for each group are presented in Table 2. Mann–Whitney *U* tests (corrected for multiple comparisons using the FDR correction) revealed that children and adults were largely comparable in their frequency of SCRs; however, adults had significantly more SCRs during the synchronous Free movement period compared to children (*U* = 50, *p* = .002, *r*_B_ = .63, BF_10_ = 11.03). All other comparisons between children and adults were not significant (all *U* > 74, *p* > .028, *r*_B_ < .452, BF_10_ = .36–2.16). This suggests strong evidence that adults had selective and increased SCR frequency during synchronous visuomotor feedback only (and anecdotal evidence the groups were similar in the remaining conditions).

**TABLE 2:** Average skin conductance responses (SCRs) frequencies for children and adults during each condition, under synchronous and asynchronous visuomotor feedback.

	Children	Adults
Baseline	5	3
Free movement		
Synchrony	6	12
Asynchrony	6	11
Structured movement		
Synchrony	6	4
Asynchrony	5	5

*Note*: There was no visuomotor feedback in the Baseline condition, and therefore no breakdown by synchrony.

##### Magnitude (strength) of elicited SCRs

The average magnitude of standardised (*z*‐scores) SCRs during the baseline, free movement, and structured movement for each group (see Table 3) were analysed using a 5 (condition: baseline, free movement synchronous, free movement asynchronous, structured movement synchronous, structured movement asynchronous) × 2 (age: children, adults) mixed ANOVA. There was a main effect of condition, *F*(4, 124) = 6.142, *p* < .001, $\eta_{p}^{2}$ = .165, BF_10_ = 534.97, no main effect of group, *F*(1, 31) = 2.314, *p* = .138, $\eta_{p}^{2}$ = .069, BF_10_ = .39, and no significant interaction *F*(4, 124) = 1.97, *p* = .104, $\eta_{p}^{2}$ = .060, BF_10_ = .81. The BF values suggest anecdotal evidence that children and adults were comparable, but decisive evidence that SCR magnitudes were higher during synchronous compared to asynchronous movement across experimental conditions.

**TABLE 3:** Average skin conductance responses (SCRs) magnitudes (raw μS with the standardised *z*‐score values in brackets) for children and adults during each condition, under synchronous and asynchronous visuomotor feedback.

	Children	Adults
Baseline	.40 (−.16)	.14 (−.74)
Free movement		
Synchrony	.50 (.14)	.28 (.18)
Asynchrony	.55 (.02)	.24 (−.05)
Structured movement		
Synchrony	.29 (−.49)	.23 (−.32)
Asynchrony	.46 (−.09)	.20 (−.35)

*Note*: There was no visuomotor feedback in the Baseline condition, and therefore no breakdown by synchrony. Values represent raw microsiemens μS, and values in brackets represent standardised *z*‐score values (see SCR analysis).

## DISCUSSION

In the present study, we explored the multisensory processes underlying embodiment for adults and young children aged 5–6 years using a full‐body virtual avatar. Participants embodied a virtual avatar that moved either synchronously or asynchronously with their own body movements while performing a self‐generated free movement period and a structured task (reaching to coloured targets). While task‐directed movements (e.g., reaching for items) have been used in related studies (Dewe et al., 2021; Keenaghan et al., 2022; Weijs et al., 2021), the current study implemented a self‐generated movement period where participants could freely and actively move the virtual body. Crucially, this offered a flexible and individual motor experience akin to the real‐world, alongside a structured period of task‐directed reaching. Participants experienced their body from a first‐person perspective alongside the useful third‐person view afforded by a virtual mirror, and lastly experienced a virtual body‐threat delivered directly at the virtual body. Embodiment was quantified via a questionnaire and psychophysiological (skin conductance responses: SCRs).

The questionnaire data revealed both children and adults report increased feelings of ownership (i.e., feeling that the body is one's own) and agency (i.e., feeling in control of one's body) over a full virtual body during synchronous visuomotor feedback. This suggests our FBI was successfully elicited under conditions of visuomotor synchrony (i.e., when the virtual body moved in synchrony to the participants own body movements) compared to visuomotor asynchrony (i.e., incongruent movement) for both age groups. The findings support a wealth of previous literature that shows adults and children embody a virtual body (or limb) which moves synchronously with their own movements (Cowie et al., 2018; Dewe et al., 2021; Slater et al., 2010; Weijs et al., 2021) yet here, we extend this effect to younger children, aged 5–6 years, for a full virtual body.^3^ Overall, the present study suggests that children as young as 5 years of age are as sensitive as adults to visuomotor synchrony cues in establishing an explicit sense of self. This is consistent with the early foundations of visuomotor synchrony detection (Rochat, 1998) and mirror self‐recognition (Lewis, 2011) which we know are early features of childhood; and with previous studies showing that visuotactile cues underpin an explicit sense of bodily awareness by 4–5 years (Cowie et al., 2013). Here we extend the finding to specifically probe how visuomotor synchrony underpins the sense of bodily self in mid‐childhood. The result also supports previous findings of a matured visuomotor pathway of the bodily self for children aged 8–12 years (Weijs et al., 2021), extending this to lower ages.

However, our findings of an adult‐like preference for visuomotor synchrony for feelings of agency (i.e., children show increased agency during synchronous visuomotor feedback) are somewhat inconsistent with related studies. For example, children at 5 years old embody a virtual body if it moves asynchronously to their own movements (Keenaghan et al., 2022) and children aged 8–12 years are less affected by visuomotor synchrony, and do not show increased agency for synchronous movement conditions (Weijs et al., 2021). The reasons for these discrepancies are less clear, but some tentative suggestions are put forward relative to different task designs. In previous studies, participants followed instructions or directed action (Keenaghan et al., 2022; Weijs et al., 2021) yet the current study included a period of free, self‐generated action. Research suggests that self‐generated action results in greater agency, perceptual awareness and VR immersion (Choi et al., 2016; Tsakiris & Haggard, 2005), and so the self‐generated action in each visuomotor condition may have modulated feelings of agency. In addition, regarding the study by Keenaghan et al.'s (2022), children were required to embody different‐sized virtual avatars and make body size estimations using tactile cues, which might have required greater attentional weighting (to complete the task) compared to visuomotor cues. In contrast, the children in our study viewed one virtual body, which moved freely during synchronous and asynchronous visuomotor conditions. Therefore, participants may have had greater attentional weighting (or less tolerance for ambiguity) on the synchronicity of visuomotor signals.

In relation to group differences, there was no difference between children and adults on agency ratings (anecdotal evidence for H_0_). However, children revealed higher ownership ratings for the synchronous visuomotor condition compared to adults. This supports previous research with the RHI that shows children have increased ownership (indicated by questions or proprioceptive drift: i.e., an index of hand localization) for a rubber hand during synchronous visuotactile conditions or congruent posture compared to adults (Cowie et al., 2013, 2016, 2022; Filippetti & Crucianelli, 2019; Gottwald et al., 2021). This is consistent with the high visual reliance suggested by previous developmental literature on hand localisation (see Introduction; Nardini et al., 2013; von Hofsten & Rösblad, 1988), extending it to a whole virtual body and suggesting that children have a strong propensity to feel increased levels of ownership for seen body parts. This is also consistent with data from Weijs et al. (2021), but extends it to a younger age group.

Importantly, there were no reliable differences between children's and adults' responses to the Control question in any visuomotor condition. The BF indicates anecdotal evidence for this null hypothesis; however, it is noteworthy that most children answered correctly (‘no’) to this question (and none of the results changed when the two participants who selected yes to this question were temporarily removed). There were no differences between children and adults on the agency and ownership questions (albeit anecdotal evidence for H_0_), which suggests children were not answering highly or unreliably and were therefore comparable to adults. Overall, the questionnaire data and the majority of SCR data suggest both age groups are comparable.

Despite the questionnaire ratings showing a clear preference for visuomotor synchrony, this was not reflected in any of the SCR data. The visuomotor synchronicity of the virtual body had no effect on levels of autonomic arousal (i.e., frequency or magnitude of SCRs) to a body‐threat. This was surprising given the wealth of previous research showing increases in SCRs for body‐threats delivered to a virtual body/body part under synchronous visuomotor conditions (e.g., Ehrsson, 2007) or for body‐threats delivered to a rubber hand under synchronous visuotactile conditions (Armel & Ramachandran, 2003). However, this lack of an effect is not so surprising given that the efficacy (frequency) and magnitude (strength) of body‐threat SCRs were lower than expected. The body‐threat (snowballs) in the visuomotor synchronous condition generated a threat‐SCR for 60% (adults) and 56% (children) of participants. However, similar studies (adult samples) found threat‐SCRs were generated in 70–100% of participants for body‐threats, and 48–68% for baseline/non‐body threat stimuli (Braithwaite et al., 2017, 2020; Dewe et al., 2018).

Similarly, the average body‐threat SCR magnitudes were lower than expected: with .50 μS (microsiemens) for children and .14 μS for adults in the synchronous visuomotor condition. In contrast, previous studies with adults typically report SCR amplitudes of 1.28–1.69 μS for body‐threats and .54–.94 μS for baseline/non‐body threats (Braithwaite et al., 2017, 2020). Interestingly, findings in this study are comparable to the Weijs et al. (2021) study of 8–12‐year‐olds, who report average SCR amplitudes of .27–1.14 μS for a body‐threat, and who also found no effect of visuomotor synchrony or a difference between children and adults. We suggest the threats we presented, while carefully calibrated so as not to be too shocking to children, were far less threatening than stimuli used in these other paradigms, and perhaps not strong enough to elicit SCRs of the magnitude commonly observed in ‘typical’ body‐threat SCR research.

While previous literature has predominantly focused on SCRs for body‐threats, we also explored the average frequency and magnitude of SCRs during the free and structured (reaching) movement periods.^4^ To our knowledge, this is the first study to measure autonomic arousal during such a free movement period in VR with children. It should be noted that SCRs can be affected by ‘gross’ movement and may therefore be elevated due to movement artefacts (Boucsein, 2012). While little research has explored VR movement or movement artefacts on SCR components of the electrodermal activity (EDA) complex, movement tasks may not necessarily account for observable differences between experimental conditions. However, we sought to control for this. Based on recommendations (Boucsein, 2012; Braithwaite et al., 2013), we (i) recorded SCRs from the site using additional adhesive tape wrapped around the fingers and to the forearm (with slack to allow movement), (ii) used relatively minor body movements as participants had to remain seated at all times, (iii) used controlled limb movements during the structured movement reaching task where participants moved one limb at a time, (iv) ensured movement periods were consistent across age groups and completed under synchronous and asynchronous visuomotor feedback, (v) compared the free and structured movement periods to a baseline period (no movement or VR) for each individual, and (vi) standardised SCR magnitudes for each condition so the effects of one condition were considered relative to the individual's overall level of background arousal in all experimental conditions.

While general increases in the frequency or magnitude of SCRs might have therefore been expected for all movement periods, we did not observe any differences in SCRs (frequency or magnitudes) between age groups, or across conditions. The exception, however, is that adults demonstrated a higher frequency of SCRs during the free movement synchronous condition compared to children. There were no group differences in the remaining conditions: the asynchronous free movement period, the synchronous or asynchronous structured movement periods or the baseline. As seen in Table 2, children had consistent SCR frequencies for all conditions, while adults had a peak of SCR frequency during the free movement conditions. This is intriguing. It could suggest adults show heightened arousal during periods of self‐generated free movement (both synchronous and asynchronous visuomotor feedback). This effect could be driven by increased movement during this stage (e.g., movement artefacts) however this is unlikely since movements were still limited (i.e., participants remained seated) and adults' SCR frequencies were lower (and the same as children) during the synchronous and asynchronous structured movement phrase. This comparison goes beyond the scope of our study, but the role and implication of different motor actions (i.e., self‐generated or directed) on arousal is worth considering in future studies.

Similarly, no differences in SCRs (frequency or magnitudes) were observed between the baseline (no movement) condition with any of the free or structured movement conditions for adults or children. This is notable because if the data had been disrupted by movement artefacts, we would expect to see such differences between these conditions. It suggests that measures of autonomic arousal did not differ when participants were sitting still, reaching for targets, or when moving freely (note: all while sitting, and when recommended standardisation techniques were applied).

### Limitations and future work

A limitation of the current study is that embodiment was quantified via only two questions (ownership and agency). It would be beneficial to explore the components of embodiment more thoroughly with more questions (Gonzalez‐Franco & Peck, 2018; Peck & Gonzalez‐Franco, 2021), however, it is difficult to do this with young children, whose attention span is limited. What might be useful in the future is to instead ask the questions after each movement condition, to explore differences in user‐experience per each movement condition.

A second limitation is the low potency of the body‐threat in eliciting an SCR. Previous research typically uses highly negative body‐threats which are not suitable for children. The body‐threat in the current study was designed to be appropriate and not traumatising for young children. However, it is likely the snowball threats were not ‘threatening’ enough, or visible long enough (2.80 s) to reveal potential differences between conditions. In fear conditioning studies with young children, stimuli should be ‘modestly aversive’ and no more than 7.5 min in duration (Gao et al., 2010) – much longer than in the current study. Future studies could develop the potency or duration of the body‐threat to explore how the components of autonomic arousal develop in childhood.

The concept of embodiment is arguably not merely the act of reflecting on one's own body and its location but may underpin cognition more broadly. For example, bodily weight influences perception of distance (Witt et al., 2004); bodily warmth increases interpersonal warmth (Williams & Bargh, 2008). Closer to the manipulations of the present study, we know that the form of a real (Allen et al., 2023) or virtual (Farmer et al., 2014) body can change one's decisions and judgements. While this ‘embodied cognition’ approach is not without criticism (Goldinger et al., 2016), it may be pertinent for future studies to consider how embodying a virtual body which *moved* differently to one's own might affect one's broader cognitive styles or judgements, including in children.

## CONCLUSION

The present student is one of the first to explore how young children (aged 5–6 years) and adults embody a virtual avatar depending on visuomotor feedback. Alongside a structed reaching task, participants were given the opportunity to move the body freely and undirected. Collectively, the findings highlight the importance of visuomotor synchrony for feelings of embodiment (ownership and agency) in both adults and children. Specifically, children at 5 years of age actively use visuomotor information to inform their embodiment experiences, as do adults. Children are comparable to adults in relation to their SCRs (frequency and magnitude) for all conditions (i.e., baseline, free movement, structured movement, and the body‐threat). Together, the study highlights the importance of visuomotor synchrony for children's embodiment of a virtual avatar from 5 years old. Moving a virtual body did not affect autonomic responding in any condition, for either group (there seemed to be no issue with movement artefacts). Virtual body‐threats, at least in the current paradigm, were not a reliable indicator of embodiment. This work signals the significant potential of integrating embodied visuomotor VR experiences with children.

## AUTHOR CONTRIBUTIONS

**Hayley Dewe:** Conceptualization (equal); data curation (equal); formal analysis (lead); investigation (supporting); methodology (equal); project administration (supporting); supervision (supporting); writing – original draft preparation (lead), writing – review & editing (equal). **Oscar Sill:** Conceptualization (equal); investigation (lead); methodology (equal); software (supporting); visualisation (lead); writing – original draft preparation (supporting); writing – review & editing (equal). **Simon Thurlbeck:** Methodology (equal); resources (supporting); software (lead); writing – review & editing (equal). **Robert W. Kentridge:** Conceptualization (equal); funding acquisition (supporting); methodology (equal); resources (supporting); software (supporting); supervision (lead); writing – review & editing (equal). **Dorothy Cowie:** Conceptualization (equal); data curation (equal); funding acquisition (lead); methodology (equal); project administration (lead); resources (lead); supervision (lead); writing – review & editing (equal).

## CONFLICT OF INTEREST STATEMENT

The authors declare that they have no conflicts of interest with respect to their authorship or the publication of this article.

## Supporting information


Video S1.

## Data Availability

In line with open‐science protocols, the data that support the findings of this study and the Supplementary materials (Procedure Video) will become openly available here through the OSF, https://osf.io/hx5qs/?view_only=a62d719502a640be9eee306c23c798ca.

## Footnotes

^1^
For dynamic stimuli, often a longer, more liberal latency window is used (e.g., Braithwaite et al., 2020; Dewe et al., 2016; Dewe et al., 2018). We opted for an 8‐s window to ensure we captured any / all significant changes in arousal tied to the threat (i.e., arousal near the end of the stimulus presentation, where snowballs seemingly ‘hit’ or ‘passed through’ the body).

^2^
We also re‐analysed the data with two participants (children) removed who scored a 6 (maximum ‘yes’ response) to this question during one or both movement conditions but all of the results remained the same, and so they were kept in the sample.

^3^
While Dewe et al. (2021) included children aged 4–5 years old, the sample comprised a larger age with a mean age of 9. This study focuses on 5–6 years specifically; using a full virtual body (Dewe et al., 2021 used a virtual hand).

^4^
We do not consider SCRs during this period as non‐specific (or non‐event related) events given the stimulating nature of the conditions and virtual environment (e.g., the mirror).
